# The prevalence of and factors associated with inclusion of non-English language studies in Campbell systematic reviews: a survey and meta-epidemiological study

**DOI:** 10.1186/s13643-018-0786-6

**Published:** 2018-08-23

**Authors:** Lauge Neimann Rasmussen, Paul Montgomery

**Affiliations:** 1 The Danish Medicines Council, Copenhagen, Denmark; 2 Department of Social Policy, Sociology and Criminology, University of Birmingham, Birmingham, UK

**Keywords:** Systematic reviews, Language bias, Location bias, Country bias, Campbell Collaboration

## Abstract

**Background:**

Studies published in languages other than English are often neglected when research teams conduct systematic reviews. Literature on how to deal with non-English studies when conducting reviews have focused on the importance of including such studies, while less attention has been paid to the practical challenges of locating and assessing relevant non-English studies. We investigated the factors which might predict the inclusion of non-English studies in systematic reviews in the social sciences, to better understand how, when and why these are included/excluded.

**Methods:**

We appraised all Campbell Collaboration systematic reviews (*n* = 123) published to July 2016, categorising each by its language inclusiveness. We sought additional information from review authors via a questionnaire and received responses concerning 47 reviews. Data were obtained for 17 factors and we explored correlations with the number of non-English studies in the reviews via statistical regression models. Additionally, we asked authors to identify factors that support or hinder the inclusion of non-English studies.

**Results:**

Of 123 reviews, 108 did not explicitly exclude, and of these, 17 included non-English language studies. One factor correlated with the number of included non-English studies across all models: the number of countries in which the members of the review team work (*B*-value = 0.56; SE *B* = 0.24; 95% CI = 0.07–1.03; *p* = 0.02). This indicates that reviews which included non-English studies were more likely to be produced by international review teams.

Our survey showed a dominance of researchers from English-speaking countries (52.9%) and review teams consisting only of team members from these countries (65.9%). The most frequently mentioned challenge to including non-English studies was a lack of resources (funding and time) followed by a lack of language resources (e.g. professional translators).

**Conclusion:**

Our findings may indicate a connection between the limited inclusion of non-English studies and a lack of resources, which forces review teams to rely on their limited language skills rather than the support of professional translators. If unaddressed, review teams risk ignoring key data and introduce bias in otherwise high-quality reviews. However, the validity and interpretation of our findings should be further assessed if we are to tackle the challenges of dealing with non-English studies.

**Electronic supplementary material:**

The online version of this article (10.1186/s13643-018-0786-6) contains supplementary material, which is available to authorized users.

## Background

Studies published in languages other than English are often neglected when research teams conduct systematic reviews. A health technology assessment of 300 randomly sampled systematic reviews published by the Cochrane Collaboration [CC], for example, found that such studies (hereafter referred to as non-English studies) were openly excluded in more than one quarter (27%) of the reviews, while more than one third of the reviews (35%) did not state explicit language criteria. In 39% of the reviews, authors explicitly searched for non-English studies, with only 15% of all reviews including any of them [1]. Within health sciences, the relevance of non-English studies is often discussed as a question of internal validity: whether non-English studies are likely to increase or decrease bias in reviews. Hence, researchers focused on the scientific necessity to include, or to justify excluding, such studies while paying less attention to the equally important practical challenges in locating and assessing relevant non-English studies.

One stream of research has debated the risks of bias of excluding non-English studies by assessing the research designs and reporting standards of non-English and English language publications. Some studies have found that English language studies have better study design standards or higher report completeness rates than non-English studies [2, 3], while others have found no significant differences [4–7]. These divergent findings might be due to differences in sampling strategies and choice of indicators, as illustrated by one study which suggested that some non-English language publications scored better on some indicators for reporting standards and worse on others in comparison with English language studies [8].

Another stream of research has approached this debate by analysing how language inclusion influences effect estimates in meta-analyses [7, 9, 10]. A review of 50 meta-analyses found that including non-English studies influenced effect estimates in more than half of the meta-analyses: in five cases, estimates became more positive, and in 16, less positive, while the precision of the effect estimates generally decreased [2]. Egger et al. [4], looking at reports of randomised controlled trials (RCTs) conducted in German-speaking countries, found that between 1985 and 1995 authors were more likely to report their findings in English language journals when their results were statistically significant and increasingly less likely to publish in German language journals. This suggests that non-English studies are important to include to avoid bias in reviews.

The Cochrane Handbook acknowledges the risk of bias in reviews containing exclusively English language studies and somewhat vaguely recommends ‘case-by-case’ decisions concerning the inclusion of non-English studies [11]. Similarly, the methodological guidelines for Campbell Collaboration [C2] reviews warn against the risk of language bias and encourages authors not to restrict by language [12]. Other than a statement in the C2 guidelines that the removal of language restrictions in English language databases is not a sufficient substitution for searching non-English databases, neither CC nor C2 provides any practical advice to review authors on how to deal with non-English studies. The lack of concrete advice and guidelines is problematic because non-English studies have been shown to be more cumbersome for researchers to identify than English language studies. Research databases, for example, are less rigorous in their inclusion and indexing of non-English studies [13–15]. Searching specialised non-English language databases using search terms in the appropriate language might alleviate this problem [16, 17], but researchers are still limited by their own language skills or their ability to pay for the services of professional translators. For these reasons, reviewers commonly report that it is costly and time-consuming to include non-English studies and use this to justify a priori exclusion [18, 19]. Noteworthy for the present study, the role of non-English studies appears to be largely unassessed within the social sciences [20] where publication channels are more prone to publication biases [21, 22].

In short, the debate about non-English studies in systematic reviews is not only about the internal validity of the included studies, but also the challenges involved in accessing potentially relevant studies in any language. Any strategy for addressing these issues must be based on an understanding of how, when and why non-English studies are included or excluded from reviews in practice.

### Objective

This study sought to identify and explore factors that might predict the inclusion in or exclusion from systematic reviews of studies that are in languages other than English. It also sought to extend the investigation of non-English study inclusion from the health sciences to the social sciences.

## Methods

The systematic reviews published by the Campbell Collaboration constitute a relevant sample for our focus on non-English studies in the social sciences. As of July 2016, Campbell had published 123 unique reviews organised within five thematic review groups: Crime and Justice, Education, International Development, Social Welfare, and Knowledge Translation and Implementation.

Campbell states that it represents the work of a diverse group of people aiming to build a ‘world-library of systematic reviews’ [23]. Like Cochrane, Campbell seeks to ensure the quality of its reviews through the enforcement of minimum standards and peer-reviewing processes [12, 18]. Campbell’s global ambition and the institutional support it offers to review teams means that its library comprises a sample of systematic reviews with a reasonable degree of comparability. This allowed us to systematically analyse the reviews, their critical appraisal process and their success in including relevant non-English studies.

A protocol for this study was developed in advance and agreed by a panel at the University of Oxford’s Centre for Evidence-Based Intervention, in the Department of Social Policy and Intervention.

### Language inclusiveness categories

We categorised Campbell reviews according to their level of inclusion of non-English studies. Reviews that excluded non-English studies with an explicit justification in the research question or research objectives were categorised as *EL-justified* (i.e. *English language-justified*), while those that excluded non-English studies without justifications were categorised as *LOE-restricted* (i.e. *languages other than English-restricted*). Reviews that did not explicitly exclude non-English studies were categorised as *LOE-open* unless they successively included non-English studies, in which case they were *LOE-inclusive*. Finally, reviews that did not state language criteria were assumed to be *LOE-open*, an assumption tested in the statistical analysis.

### Data extraction

We developed a data extraction sheet mirroring the Campbell review template for our analysis [24] to collect data on the factors that might correlate with the number of included non-English studies (see Additional file 1). One author (LNR) conducted the data extraction and the following coding. In cases where reviews deviated from the C2 template (e.g. that by Lum et al. [25]), sections in the given C2 review that seemed likely to contain the relevant data were read and data extracted according to the pre-specified extraction sheet, but no reviews were read in full due to resource constraints.

Abstracts were assessed to determine if they included research questions that stated a geographical focus on predominantly English-speaking countries (i.e. USA, UK, Ireland, Australia and New Zealand), which could lead to categorising the review as *EL-justified*. The reliability of this procedure depends upon the review teams’ compliance with the Preferred Reporting Items for Systematic Reviews and Meta-analyses (PRISMA) standards, which advises authors to formulate research questions using the PICO format (i.e. identifying participants, interventions, comparators and outcomes) [26, 27]. Therefore, we also extracted any information about geographical limitations that were stated in the research objectives, as a way to identify when reviews were *EL-justified*.

Data were collected on the following: characteristics of the review team (number of authors; author institutions; number of different author working countries) and the systematic review (Campbell review group; publication year; inclusion criteria). In cases where a Campbell review was co-registered with the Cochrane Collaboration, this was noted, as co-registered reviews might enjoy greater institutional support during the critical-appraisal process than reviews only registered with Campbell. We also coded additional factors covering the search strategy (number of data sources sought; search terms languages; whether experts were contacted) and the flow of studies during the review process (studies located, screened, full-text assessed, included and meta-analysed). Finally, the number of non-English studies that was included in each review was estimated by counting the number of non-English titles in the list of included studies of each review.

### Author questionnaire

Having extracted the majority of data from the C2 reviews, we found that some relevant factors were likely underreported to assess their importance. We therefore sent questionnaires to the review authors to inquire about factors such as the composition of their review teams (author nationalities; languages spoken) and the use of expert networks to locate studies as well as the language of applied search strings. We also asked respondents what they perceived to be the barriers and facilitators of including non-English studies. These free-text box inputs from the author questionnaire were coded iteratively and aimed to identify the challenges that review teams experience when considering, or actually including, non-English studies in Campbell reviews.

One primary author (usually the corresponding author) was invited to respond and then reminded. If needed, an invitation was sent to the entire review authorship on whom we had contact details. In cases where an author was the primary author of more than one review, a second author was prioritised. This choice was made in an effort not to overload highly productive review authors with multiple invitations. The questionnaires were completed during June and July 2016 and were followed by a consent form.

### Statistical procedures

For some results, the median is the most valid estimate of the central tendencies in the dataset and are reported when appropriate.

Three exploratory multivariate models were tested with the software, SPSS Statistics 25 for Windows, to identify factors that correlate with the number of included non-English studies in the Campbell reviews. One model analysed all the LOE-open and LOE-inclusive reviews by including the 15 factors extracted from these reviews; a second per-protocol model added the questionnaire variables (author nationalities; languages spoken); and a third model was a sensitivity analysis that excluded reviews which do not explicitly state language eligibility criteria to test the robustness of our first model’s assumption that reviews are *LOE-open* by default. Due to the explorative nature of the study and the low statistical power from the small sample, we accepted significant associations at a *p* value of 0.10 when running regressions to identify possible associations.

## Results

We included all 123 unique systematic reviews published by the Campbell Collaboration and categorised each by its language inclusiveness (Table 1).

**Table 1:** Systematic reviews categorised by inclusiveness of studies in languages other than English (LOE)

LOE-category	Number of reviews (%)
EL-justified	0 (0)
LOE-restricted	15 (12.2)
LOE-open	84^a^ (68.3)
LOE-inclusive	17^a^ (13.8)
LOE-undefined	7^a^ (5.7)
Total reviews	123 (100)

*EL-justified* reviews that exclude non-English studies with an explicit justification in the research question or research objectives, *LOE-restricted* reviews that exclude non-English studies without justifications, *LOE-open* reviews that do not explicitly exclude non-English (unless they successively include non-English studies, in which case they are *LOE-inclusive*), *LOE-undefined* reviews that do not provide a list of included studies

^a^Twenty-seven reviews did not state any explicit language eligibility criteria. Twenty-two of these belonged to the LOE-open group, two to the LOE-inclusive group and three to the group of LOE-undefined reviews

Based on analysis of the abstracts and research objectives of the 123 reviews, none focused solely on English-speaking countries; hence, none qualified as *EL-justified*. Fifteen (12.2%) reviews explicitly stated that they excluded non-English studies and are therefore categorised as *LOE-restricted*. The most common justifications for language exclusion were resource constraints (five cases) and lack of policy relevance outside English-speaking countries (three cases). In one case, authors mentioned that possibly relevant German and French language studies had been located but not assessed. In seven cases, no comments were made to justify the language restrictions.

Of the 123 reviews, 108 (87.8%) indicated that they were either open to studies in languages other than English (*n* = 81) or reported no language criteria (*n* = 27). Among these, we categorised 84 (68.3%) as *LOE-open*, 17 (13.8%) as *LOE-inclusive* and the remaining 7 (5.7%) as *LOE-undefined*, because they did not provide a list of included studies. Thirty-nine non-English studies were included in the LOE-inclusive reviews of which nine reviews contained a single non-English study, six contained two to four non-English studies, and two reviews contained respectively six and seven non-English studies. The publication languages were Spanish (13), French (11), German (5), Portuguese (5), Italian (2), Swedish (2) and Norwegian (1).

Authors of two reviews declined to participate in the questionnaire arguing that non-English studies were not relevant to the given review, or making reference to a lack of time. Responses cover 47 (38%) of the 123 reviews. Assessing differences in median figures on selected variables (Table 2), there is little indicating that questionnaire responders and non-responders authored substantially different systematic reviews. If valid, the answers of responders can be generalised to non-responders.

**Table 2:** Comparison of reviews based on responders and non-responders of questionnaire

	Response from review author (*n* = 47)	No response from review author (*n* = 76)
Publication year	2013	2012
Author numbers	4	4
Language criteria^a^	1	1
Number of searched sources	23	21
Number of included studies	13	17

All figures are reported as medians

^a^For the language criteria variable, the value of 1 equals language openness, while the value of 0 equals the explicit restriction to English publications (LOE-restricted)

In several cases, questionnaire respondents expressed uncertainty about their co-authors’ language abilities, and some questions (those regarding the use of expert networks and language of applied search terms) prompted so vague or incomplete answers that we deemed the variables unreliable and dropped them from our analysis.

### Authorship and review characteristics

We analysed language inclusiveness based on the primary subject area of each review, using the Campbell Collaboration Review Groups as indicators of subject area (Table 3). Eleven of the 15 reviews that excluded non-English studies without justifications (i.e. *LOE-restricted*) were in Crime and Justice. Reviews that fell under the purview of the Social Welfare group represented almost half of the reviews in our study (*n* = 60), but only around 8.3% (*n* = 5) included non-English studies. International Development contained more than half of the included non-English studies (*n* = 21), although the group represents a minority (around one fifth or *n* = 25) of the total reviews. In Education, there were almost as many reviews that included non-English studies (*n* = 18) as in International Development, but the former represents a slightly larger proportion of the total reviews (*n* = 29).

**Table 3:** Differences in language inclusiveness based on subject area (i.e. Campbell Collaboration Review Group)

Language category	Number of reviews in each subject area (%)			
	Crime and Justice^a^	International Development^a^	Education^a^	Social Welfare^a^
LOE-restricted	11	0	3	1
	(24.4)	(0.0)	(10.3)	(1.7)
LOE-open	29	18	20	54
	(64.4)	(72.0)	(69.0)	(90.0)
LOE-inclusive	5	7	6	5
	(11.1)	(28.0)	(20.7)	(8.3)
Total reviews^b^	45 (36.6)	25 (20.3)	29 (23.6)	60 (48.8)
Number of non-English studies included^b^	13 (33.3)	21 (53.8)	18 (46.2)	8 (20.5)

^a^The *Knowledge Transfer and Implementation* group has been dropped from the table as it contains only a single *LOE-open* review

^b^Some reviews, and their included non-English language studies, belong to more than one of the thematic groups and are therefore counted more than once

Thirty-nine (31.7%) C2 reviews were also registered with the CC, but only two of the co-registered reviews included non-English studies. Thus, 15 of 17 (88.2%) LOE-inclusive reviews were published exclusively by the Campbell Collaboration and accounted for 37 of the 39 (94.9%) non-English studies included in the total sample of C2 reviews.

Each review involved between four and five authors, who tended to be affiliated with two or three different institutions working within the same country (Table 4). Based on results of the author questionnaire, the review teams usually represented one or two nationalities and one or two languages, although teams that conducted LOE-inclusive reviews tended to speak four languages. However, in several cases, questionnaire respondents expressed some uncertainty about their co-authors’ language skills.

**Table 4:** Author and systematic review characteristics

Variables	LOE-restricted reviews (*n* = 15)	LOE-open reviews (*n* = 84)	LOE-inclusive reviews (*n* = 17)
Author characteristics			
Number of authors			
Mean (SD)	4.2 (1.2)	4.6 (2.6)	4.9 (2.1)
Median	4	4	5
Number of institutions represented by authors^a^			
Mean (SD)	2.3 (1.0)	2.4 (1.6)	2.8 (1.9)
Median	2	2	3
Number of different working countries represented by authors^a^			
Mean (SD)	1.3 (0.6)	1.3 (0.7)	1.8 (1.3)
Median	1	1	1
Questionnaire variables	(*n* = 6)	(*n*=30^b^)	(*n*=9^b^)
Authors’ languages			
Mean (SD)	1.3 (0.5)	3.2 (2.2)	4.2 (2.5)
Median	1	2	4
Authors’ nationalities			
Mean (SD)	2.0 (0.9)	2.1 (2.2)	2.7 (1.2)
Median	2	2	2
Methodological inclusion criteria			
RCTs (%)	8 (53.3)	31 (36.9)	4 (23.5)
Quasi-experiments (%)	3 (20.0)	48 (57.1)	12 (70.6)
Non-experiments (%)	3 (20.0)	5 (6.0)	1 (5.9)
Unclear (%)	1 (6.7)	0 (0.0)	0 (0.0)
Scope of search strategy			
Languages of search terms			
Mean (SD)	1 (0.0)	1.3 (1.1)	1.4 (1.5)
Median	1	1	1
Databases, registers and journals searched in			
Mean (SD)	34.6 (36.8)	25.3 (23.0)	34.4 (22.0)
Median	26.0	21.5	26.0
Experts contacted			
Yes (%)	9 (60.0)	63 (75.0)	14 (82.4)
No (%)	6 (40.0)	21 (25.0)	3 (17.6)

The seven LOE-undefined reviews are not included in the regression analyses. The total number of reviews included in the analysis is thus 116

^a^In 35 reviews, one or more author institutions were not reported accounting for 18.4% of the total 570 authors

^b^Two respondents did not give complete answers about the nationalities and languages of their review team; thus, there was one missing value for *LOE-open* and one for *LOE-restricted*

*N/A* Not Applicable, i.e. no methodological inclusion criteria were unclear

Reviews that included non-English studies were more likely to accept quasi-experimental designs (70.6%) in addition to RCTs, while reviews that excluded non-English studies without justifications (*LOE-restricted*) and those that did not explicitly exclude non-English studies (*LOE-open*) were less likely to accept quasi-experimental designs (20% and 57.1%, respectively).

The authors of reviews that included non-English studies were more likely to contact experts to identify relevant studies (82.4%), compared to the authors of reviews in the other two categories (*LOE-restricted* = 60%; *LOE-open* = 75%).

Review teams typically searched between 21 and 26 databases, registers and journals, but rarely with non-English search terms. Only in 11 reviews did authors apply search terms in Spanish (8), Swedish (7), Portuguese (4), French (3), Norwegian (3), Danish (2) and Arabic, Chinese, Dutch, German, Italian and Russian (1 each).

**Fig. 1:**
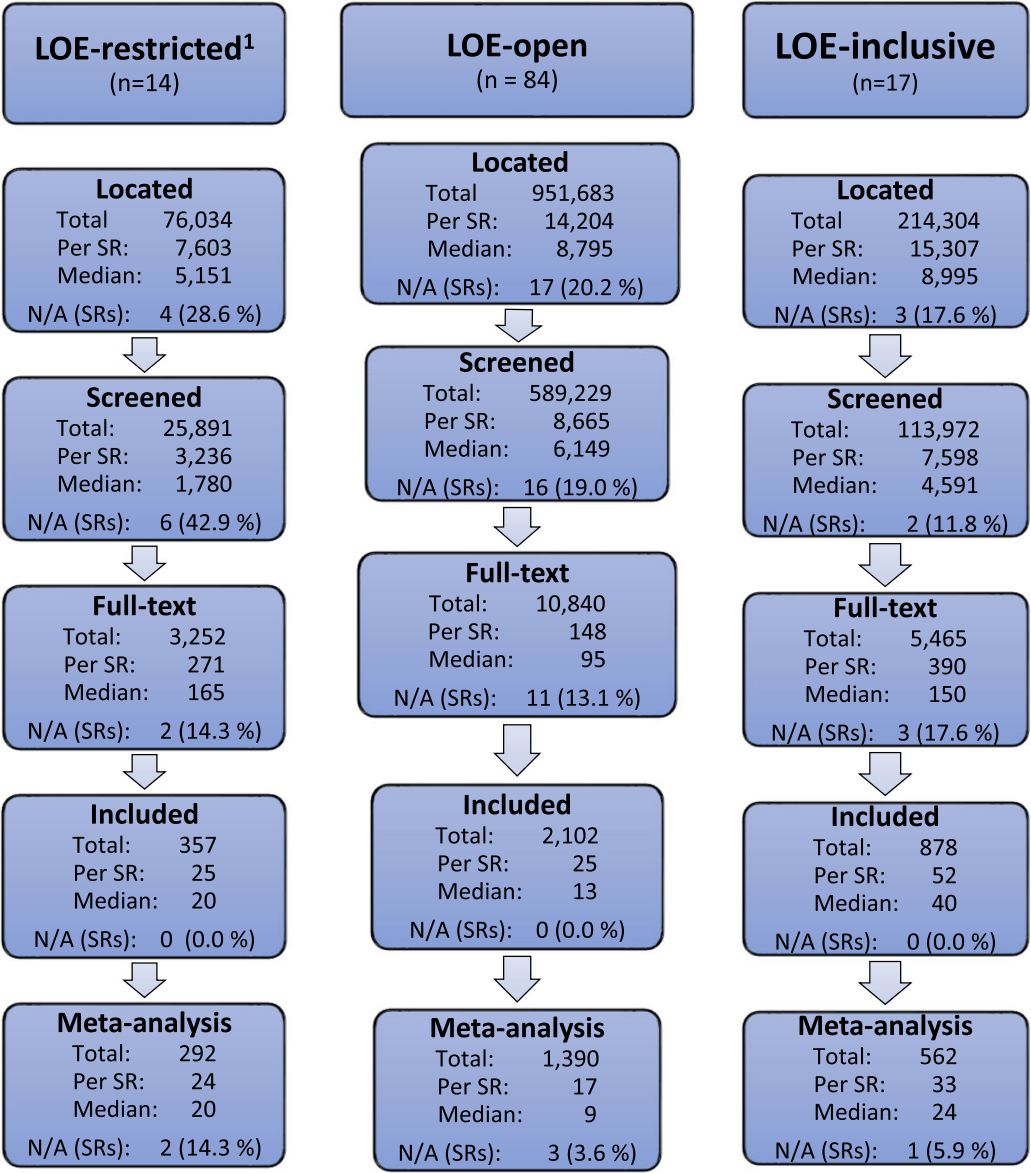
Synthesised study flow diagrams based on the sample of systematic reviews published by the Campbell Collaboration, excluding those seven reviews that did not provide a list of included studies [28]

Figure 1 outlines the pooled flow of studies following the PRISMA-diagram framework [26]. Each diagram represents one language category and lists the mean, median and total number of studies located, screened, full-text assessed, included and meta-analysed throughout the review process.

The figure reveals that reviews that excluded non-English studies without justifications (*LOE-restricted*) located (median = 5151) and screened (median = 1780) substantially fewer studies than reviews that did not explicitly exclude non-English studies (*LOE-open*: respective medians = 8795; 6149) and reviews that ultimately included non-English studies (*LOE-inclusive*: respective medians = 9995; 4591). Reviews that included non-English studies (*LOE-inclusive*) assessed more full text studies (median = 150) than reviews that did not exclude non-English studies (*LOE-open*: median = 95) though assessing less than those that excluded non-English studies (*LOE-restricted*: median = 195). Noticeably, the reviews that included non-English studies also included at least twice as many studies (median = 40) than the other two categories (*LOE-open*: median = 13; *LOE-restricted*: median = 20). However, the former review category tended to discard far more studies from meta-analysis compared to the other categories. The assessment of the *LOE-inclusive* reviews showed that 31 of the 39 non-English studies that were included in Campbell reviews were subsequently also included in meta-analyses.

Overall, the success rate from screening to including studies were 1.1%, 0.2% and 0.9% (based on medians in Fig. 1) in LOE-restricted, LOE-open and LOE-inclusive respectively—and substantially lower if one calculates success rate based on the number of located studies or the number of included non-English studies for LOE-inclusive reviews.

### Regression analyses

The three exploratory regression models explain between 0.37 and 0.59 of the variation in the data (Table 5). In the first model (*p* = 0.05), with data from the survey of included reviews, one factor (*number of different working countries represented by authors*) was significant with a *B*-value of 0.56 (SE *B* = 0.24; 95% CI = 0.07–1.03; *p* = 0.02). This suggests that when a review team included an author working in a different country than the rest of the authorship, the review was, on average, likely to include 0.56 more non-English studies. Similar, but slightly stronger, correlations between the number of different working countries and number of included non-English studies were identified in the two other models: model 2 (*p* = 0.07) with the survey data identifies a *B*-value of 0.96 (SE *B* = 0.43; 95% CI = 0.07–1.86; *p* = 0.04) and the third model (*p* = 0.09), which excluded those reviews that did not state explicit language criteria, identifies a *B*-value of 0.65 (SE *B* = 0.30; 95% CI = 0.05–1.25; *p* = 0.04).

**Table 5:** Factors correlating with the number of non-English studies included in Campbell systematic reviews

Models	*B* (CI 95%)	Sig.	Standard error	Std. coefficients (beta)
Model 1 (*n* = 101) survey model		0.05		
Constant	− 0.93 (− 2.39–0.52)	0.20	0.73	
Number of different working countries represented by authors	0.56 (0.07–1.03)	0.02	0.24	0.41
Education group	0.94 (− 0.10–1.98)	0.08	0.52	0.32
Number of included studies	0.01 (0.00–0.02)	0.06	0.01	0.34
Model 2 (*n* = 47) survey with questionnaire model		0.07		
Constant	− 4.53 (− 9.38–0.32)	0.07	2.34	
Number of different working countries represented by authors	0.96 (0.07–1.86)	0.04	0.43	0.42
Crime and Justice group^a^	3.99 (− 0.02–7.99)	0.05	1.93	0.91
Education group^a^	2.85 (− 0.31–6.02)	0.08	1.53	0.89
International Development group^a^	1.92 (0.08–3.75)	0.04	0.89	0.49
Social Welfare group^a^	2.85 (− 0.57–6.23)	0.09	1.65	0.97
Model 3 (*n* = 77) language explicit model		0.09		
Constant	− 0.82 (− 2.78–1.14)	0.40	0.94	
Number of different working countries represented by authors	0.65 (0.05–1.25)	0.04	0.30	0.48
Number of screened studies	0.00 (0.00–0.00)	0.09	0.00	− 0.55
Number of included studies	0.02 (0.00–0.03)	0.06	0.01	0.40

The independent variable is the number of non-English studies included in the systematic reviews published by the Campbell Collaboration. The variable, *number of studies meta-analysed*, was dropped from the analyses because it had an unacceptably high correlation with the variable accounting for the *number of studies included in reviews* (Pearson’s *R* = 0.85; *p* < 0.01)*.* All other variables were included as planned, but only significant variables are reported. Missing data is excluded pairwise

*R*^2^ = model 1, 0.37; model 2, 0.59; model 3, 0.40

^a^The substantial implication of belonging to any one subject area covered by C2 is illogical and is thus disregarded

Two other variables, number of included studies (model 1 and model 3) and number of screened studies (model 3), showed significant correlations with the inclusion of non-English studies. The *B*-values for the number of included studies range between 0.01 and 0.02 indicating that including 50–100 additional studies, on average, correlates with the inclusion of an additional non-English study. The correlation between the number of screened studies and number of included non-English studies in model 3 is, though significant, substantially un-interpretable at first (*B*-value = 0.00; SE *B* = 0.00; 95% CI = 0.00–0.00; *p* = 0.09). However, as standardised coefficients (beta), their magnitude (model 1: included studies = 0.34; model 3: included studies = 0.40, screened studies = − 0.55) are equivalent to the standardised coefficients of the number of author countries ranging between 0.41 and 0.48. Substantially, this could be interpreted as an indication that the more studies review teams include, the more non-English studies they are likely to include, while the more studies review teams screen, the less likely they are to include non-English studies.

To counter non-normal data distribution, all models were bootstrapped with 1000 samples. None of the models were significant after this procedure. Countering substantial multicollinearity, a simpler model with seven variables (number of authors, author institutions, author working countries, methodological criteria, sources searched, use of experts and search-term languages) was tested and again identified a positive relationship between the number of working countries represented by authors and the number of reviews that included non-English studies.

Assessing ‘author country’ more closely, we found that 52.9% of review authors worked in the USA or the UK (Table 6). In fact, 65.9% of the teams (*n* = 81) only had members working in English-speaking countries, while 34.1% (*n* = 42) had one or more members working outside an English-speaking country.

**Table 6:** Review authors’ working countries

Author countries	Number of authors	% of authors
USA	162	28.4
UK	140	24.5
Canada	38	6.7
Norway	35	6.1
Denmark	24	4.2
Australia	17	3.0
The Netherlands	13	2.3
South Africa	11	1.9
Switzerland	6	1.1
Sweden	3	0.5
Other	16	2.8
Unclear	3	0.5
Total	468	82.0
N/A	102	17.9
Total with N/A	570	99.9

*N/A* the number of authors for whom their working country is not reported in the review

### Barriers to and facilitators of including non-English studies

Unsurprisingly, authors commonly pointed to issues of cost, time and funding as crucial for the inclusion of non-English studies, as well as lack of language *resources* (Table 7). ‘Language resources’ here refers to people or services *external* to the review team (e.g. professional and volunteer translators, software translation tools and English abstracts). ‘Language skills’—the language competencies within the review teams (e.g. multilingual authors and affiliated staff)—was not experienced as a barrier, nor a facilitator, as often as language resources, but was still pointed to as the third most common barrier. Slightly more often than language skills, authors pointed to the need for training in and guidelines on how to deal with non-English studies and access to non-English specialised databases as important facilitators. Issues of bias and methodological quality were mentioned, although infrequently.

**Table 7:** Barriers to and facilitators of including non-English studies

Barriers	Number (%) of reviews that identify this as a barrier	Facilitators	Number (%) of reviews that identify this as a facilitator
Cost and time	18 (38.3)	Language resources	20 (42.6)
Lack of language resources	17 (36.2)	Funding and time	11 (23.4)
Lack of language skills	12 (25.5)	Training in and guidelines on how to deal with non-English studies	9 (19.1)
Lack of (access to) non-English specialised databases	8 (17.0)	Access to non-English specialised databases	8 (17.0)
Complacency of review authors	3 (6.4)	Language skills	7 (14.9)
Biases and low quality of non-English studies	3 (6.4)	Cochrane and Campbell cooperation	5 (10.6)
		Availability of quality non-English studies	3 (6.4)
Other	9 (19.1)	Other	6 (12.8)

*N* = 47. Due to the open-ended format of the questions on barriers and facilitators, respondents’ answers sometimes related to more than one theme. The total count of barriers and facilitators therefore added up to more than the number of respondents

## Discussion

Among the 123 reviews in our study, 108 did not exclude non-English studies a priori, and of those who did, few justified their reasons to do so*.* The relatively low prevalence of non-English studies in our sample of reviews might be somewhat underestimated by our data extraction approach, counting non-English titles in the study inclusion list. Assuming that this is not the case, the low prevalence might indicate that relevant non-English studies were not available or that the review teams failed to identify these studies. We did not assess whether relevant non-English studies had been overlooked or, if located, were excluded due to risk of bias. Overall, however, the infrequent number of non-English studies does leave some room for C2 to convincingly develop a ‘world-library of systematic reviews’ [23].

The higher acceptance of quasi-experimental designs by reviews that included non-English studies might be interpreted as an indication of lower methodological criteria thresholds. However, the relevance of studies does not depend simply on their position in the hierarchy of evidence but also on other factors such as the rigour by which they have been conducted and the contextual feasibility of research designs for a given research topic. We assumed that, by following the Cochrane Collaboration standards, the reviews published by Campbell included rigorous critical appraisal of all included studies. With this assumption, our statistical analyses did not indicate that the methodological threshold or any other step of the critical appraisal process affected whether non-English studies were included. We also did not find any indications that co-registered reviews with institutional support from both Cochrane and Campbell were more likely to include non-English studies than those published exclusively by Campbell.

Results of the author questionnaire suggested that the most obvious challenges to include non-English studies were resource constraints and, somewhat linked to this, the reliance of research teams on their own internal language skills. In this light, Fig. 1 illustrates that review teams may expect an overwhelming number of studies to screen and full-text assess when seeking to include non-English studies. To counter this challenge, we suggest two options that could lower the work load burden for C2 review teams and improve the review quality. First, teams might benefit from putting more effort into improving the specificity of their research questions and search strategies. Our regression analyses (Table 5) indicated a negative relationship between the number of studies screened and the inclusion of non-English studies, as well as a positive relationship between the number of studies included and the inclusion of non-English studies. These correlations were not consistent between our regression models; thus, the results and interpretations are somehow speculative but could suggest that authors of LOE-inclusive reviews conducted searches that more successfully than authors of LOE-open reviews balanced sensitivity and specificity. Indeed, Fig. 1 does illustrate that LOE-inclusive reviews succeed in including more relevant studies disregarding publication language than LOE-open reviews, while screening substantially fewer studies.

Second, more review teams could consider explicitly restricting their reviews to English language publications and state, as well as justify, this limitation, e.g. in abstracts, research questions, objectives and eligibility criteria. Pragmatically restricting reviews to English publications is legitimate but should be clearly acknowledged and the limitations in findings and their relevance should then be properly discussed by review teams. Future C2 guidelines could address these issues more clearly as called for by some of our questionnaire respondents.

Considering that our statistical models indicated that the composition of review teams working across countries significantly correlates with the number of included non-English studies, one can speculate whether more international review teams master more languages than less international teams and that this perhaps allows the former to pursue the identification of non-English studies more diligently. This speculation is not supported by our statistical models, which did not identify language as being of significant importance. However, the data on author languages was to some degree unreliable as questionnaire respondents expressed uncertainty about languages spoken by their co-authors. Further, the questionnaire only covered 47 of the 123 review teams, which lowers its statistical power to identify a real relationship, if one exists. The statistical power of another language variable identified by earlier research [16, 17]—the application of non-English search term in the literature search process—is also low due to the few reviews that applied non-English search terms. We therefore cannot confirm the importance of language in accessing non-English studies, nor do we have reason to reject the importance of language diversity.

An alternative interpretation of the statistical relationship between author countries and included non-English studies is that international review teams have easier, perhaps informal, access to a more diverse set of language resources than teams working within the same country. It might also be that the range of author countries is a proxy for knowledge about and access to more diverse or specific publication channels that facilitate the inclusion of non-English studies. Finally, there might be a degree of selection effect operating, whereby international review teams pick research topics with more global relevance and therefore a higher prevalence of non-English studies.

### Limitations

A main limitation of the present study relates to its exploratory nature and the statistical robustness of the findings. First, only one author (LNR) conducted the data extraction and coding, meaning there could be a degree of bias and risk of errors in the process. However, a protocol was put in place to guide the project and frequent support and supervision was given with the second author. Some caution is also warranted considering the statistical issues of non-normality, relatively high levels of multicollinearity and chances of random error when dealing with 15 to 17 factors within a relatively small dataset. Still, the relation between author countries and included non-English studies was consistent for all models, except for the bootstrapped ones, which added some credibility to the results, supported by the qualitative data. Unfortunately, the results are somewhat confounded by the 35 systematic reviews—accounting for 17.9% of the total Campbell authorship (Table 6)—that did not report exhaustively on the institutional affiliation of all review authors. Some statistical power could perhaps have been gained had we had the resources to read the 123 reviews in full, e.g. in the hope of identifying the publication languages of those individual studies that were included in the seven C2 reviews which, surprisingly, did not provide a basic list of included studies.

There are also limits to the depth of the dataset. We found few and smaller differences when comparing the three review categories (Table 4), for example in relation to the number of data sources sought. In practice, however, the number of data sources might be less relevant than which (non-English language specialised) data sources a review team searched. Perhaps the clear dominance of individual researchers based in English-speaking countries and review teams consisting only of team members in these countries reflects a partiality among publication channels for studies in English. Working country is not synonymous with the origin of authors and thereby which languages review teams might master, but it is possible that our survey did not yield sufficient, nor adequately reliable, information to identify a possible association with the number of included non-English studies. Additionally, if the low prevalence of non-English search terms is a proxy for the general rigour with which non-English studies have been pursued, the factor that we identified (authors’ working countries) might not be the most effective. Factors such as the number of search-term languages might be more important in practice if they were applied more often.

At the moment, we cannot tell to which degree the results can be extrapolated from our sample of Campbell reviews to the wider population of reviews. Knowing the differences in publication channels between social sciences and health sciences [21], and considering the substantial differences in including non-English studies between the reviews in our sample that were co-published with the CC and those published exclusively by the C2, we would encourage the replication of this study’s research design, for example with a sample of systematic reviews from the Cochrane Collaboration.

Finally, we believe new perspectives and a deeper understanding of the systematic challenges in dealing with non-English studies could be obtained by approaching the issue through more qualitative methods. Interviews with internationally experienced reviewers could, for example help map out more extensively the practical barriers and facilitators for the inclusion of non-English studies in systematic reviews. To our knowledge, such a study design would be the first of its kind on an issue that has been dominated by quantitative study designs.

## Conclusion

We investigated the factors that might predict the inclusion of studies that are in languages other than English in systematic reviews, particularly in the social sciences. We analysed all 123 systematic reviews published by the Campbell Collaboration, categorising each by its language inclusiveness. We also sought additional data from review authors and received responses from around one third of our sample.

The majority of Campbell reviews (*n* = 108) did not explicitly exclude non-English language studies, and 17 (13.8%) actually included non-English language studies. The most obvious challenge to including non-English studies, according to review authors, was cost and time. This might be a key reason for another common obstacle: review teams’ reliance only on their own language skills, rather than calling on the support of professional translators.

Overall, our sample of reviews showed a clear dominance of individual researchers based in English-speaking countries and review teams consisting only of team members in these countries, which could reflect a partiality among social science publication channels for studies in English.

Reviews which included non-English studies were more likely to be produced by review teams comprised of members working across different countries and languages. However, the reasons for this are unclear. For example, international review teams may have easier, perhaps informal, access to and/or knowledge about a more diverse set of language resources and publication channels than teams working within the same country. Or there might be a degree of selection effect in play, whereby international review teams pick research topics with more global relevance and therefore a higher prevalence of non-English studies.

This study has highlighted some of the remaining questions around language inclusiveness in systematic reviews and the unique challenges involved in locating and assessing available non-English studies. These studies might ensure the internal validity of findings, or perhaps increase external validity to the degree that reviews with non-English studies differ from those with only English language studies with respect to the location, culture and specific population groups they represent. In light of these issues, we recommend replicating our study using a wider range of reviews, for example using the Cochrane Library as a sample. Such efforts are crucial if the evidence-based movement is to succeed in becoming a global movement of people aiming to build a world library of systematic reviews.

## Additional file

Additional file 1: File containing the data extraction sheet used to collect data on the Campbell Collaboration systematic reviews. (PDF 498 kb)

## Abbreviations

EL-justified: English language-justified, i.e. reviews that exclude non-English studies with an explicit justification in the research question or research objectives
LOE-inclusive: Languages other than English-inclusive, i.e. reviews that include non-English studies
LOE-open: Languages other than English-open, i.e. reviews that do not explicitly exclude non-English studies
LOE-restricted: Languages other than English-restricted, i.e. reviews that explicitly exclude non-English studies without justifications
LOE-undefined: Languages other than English-undefined, i.e. reviews that do not provide a list of included studies
PRISMA: Preferred Reporting Items for Systematic Reviews and Meta-analyses
