# Supplementary material for: The prevalence of and factors associated with inclusion of non-English language studies in Campbell systematic reviews: a survey and meta-epidemiological study
Source: Syst Rev. 2018 Aug 23;7:129. doi: 10.1186/s13643-018-0786-6 (PMC6107944; doi:10.1186/s13643-018-0786-6)
Supplement: Supplementary file 1 — File containing the data extraction sheet used to collect data on the Campbell Collaboration systematic reviews. (PDF 498 kb) [file 13643_2018_786_MOESM1_ESM.pdf]

Supplementary material for

The prevalence of and factors associated with inclusion of non-English language studies in Campbell systematic reviews: a survey and meta-epidemiological study

Lauge Neimann Rasmussen (corresponding author), MSc Evidence-Based Social Intervention and Policy Evaluation, University of Oxford, lauge@protonmail.com

Paul Montgomery, Professor of Social Intervention at the University of Birmingham. Birmingham. UK. paul.x.montgomery@bham.ac.uk

Data extraction sheet

Data Extraction Sheet for "The Prevalence and Importance of Language Inclusive Systematic Reviews: The case of The Campbell Collaboration"

Campbell Library Website

1. **Title of systematic review:**

2. **Names of authors:**

3. **Number of authors:**

4. **Year of last update/publication:**

5. **Campbell Collaboration Coordination Group:**
   Tick all that apply.
   - [ ] Crime and Justice
   - [ ] Education
   - [ ] International Development
   - [ ] Social Welfare
   - [ ] Methods
   - [ ] Knowledge Translation and Implementation

Colophon

6. **Is the review co-registered with the Cochrane Collaboration?**
Mark only one oval.

- Yes
- No

Abstract

7. **Does the abstract focus on English-speaking countries only?**
By English-speaking countries is meant the USA, UK, Ireland, Australia and New Zealand.
Mark only one oval.

- Yes
- No
- Unclear

8. **If yes or unclear, specify the framing:**

Objective

9. **Is the research question limited by geography or nationality?**
Mark only one oval.

- Yes
- No

10. **If yes, specify:**

Methods section

11. **What types of research designs are to be included?**
Tick all that apply.

- RCTs, quasi-RCTs and cluster RCTs
- Quasi-experiments
- Non-experiments
- Unclear
- Other:

12. **Explicit LOE-related inclusion/exclusion criteria:**
Mark only one oval.

- ◯ Language restricted
- ◯ Language open
- ◯ Not stated
- ◯ Other: ____________

13. **Specify LOE-related inclusion/exclusion:**

____
____
____
____
____

Search Process

14. **Which databases, registers and journals were explicitly searched?**
Copy paste all sources listed in the review.

____
____
____
____
____

15. **Were informal/expert contacts utilised when searching for relevant studies?**
Mark only one oval.

- ◯ Yes
- ◯ No

16. **If yes, how was the contact strategy elaborated?**
Tick all that apply.

- ☐ Not elaborated
- ☐ Number of informal/expert contacts
- ☐ Names of informal/expert contacts
- ☐ Institutional affiliation of informal/expert contacts
- ☐ Contact information provided
- ☐ Other: ____________

17. **Language of search terms:**
Tick all that apply.

- ☐ English
- ☐ Other: ____________

18. **If non-English search terms were claimed, were they available in each language?**
Mark only one oval.

- Yes
- No
- N/A
- Other: ____________________

Results

19. **How many studies were identified/located during the initial search?**

____

20. **How many studies had their titles and abstract screened?**

____

21. **How many studies had their full-text assessed for inclusion?**

____

22. **How many studies were included in the systematic review?**

____

23. **How many studies were included in the meta-analyses?**

____

24. **How were LOEs said to be critically appraised?**
Tick all that apply.

- [ ] Not adressed
- [ ] Via software translation tools (e.g. Google Translate)
- [ ] Via a non-English translator (professional, volunteer, other)
- [ ] By non-English speaker(s) in the reviewer group
- [ ] Unclear
- [ ] Other: ____________________

Discussion and Conclusion

25. **Copy-paste any comments and reflections made about the language exclusion/inclusion of the study:**

26. **Copy-paste any comments and reflections made about the geographical limits of the study?**

27. **For future updates, do the reviewers explicitly plan/recommend to expand the language sensitivity?**

Mark only one oval.

- Yes
- No

28. **If yes, how? (Copy paste)**

Appendices

29. **Based on titles, how many LOE studies are present in the list of included studies?**

Author Characteristics

From colophon, appendix, or alternatively protocol.

30. **Institutional affiliations of authors:**
Write the institution of each author separately.

31. **Authors countries:**
Write the country of each author separately.

32. **Reviewers own comments:**

Powered by
Google Forms
